# Ground beetles in city forests: does urbanization predict a personality trait?

**DOI:** 10.7717/peerj.4360

**Published:** 2018-02-20

**Authors:** Wiebke Schuett, Berit Delfs, Richard Haller, Sarah Kruber, Simone Roolfs, Desiree Timm, Magdalena Willmann, Claudia Drees

**Affiliations:** Zoological Institute, Biocenter Grindel, Universität Hamburg, Hamburg, Germany

**Keywords:** Anthropogenic change, Boldness, Carabid, Density, Environmental driver, Exploration, Invertebrate, Novel environment, Open field, Temperature

## Abstract

**Background:**

Urbanization leads to substantial changes in natural habitats with profound effects on wildlife. Understanding behavioural responses to such environmental change is essential for identifying which organisms may adapt, as behaviour is often the first response to altered conditions. Individuals in more urbanized habitats may be expected to be more exploratory and bolder than their conspecifics in less urbanized habitats as they may be better able to cope with novel challenges.

**Methods:**

In a two-year field study we tested ground beetles from differently urbanized forests for their exploratory behaviour (in a novel environment) and their risk-taking (death-feigning). In total, we tested ca. 3,000 individuals of four forest-dwelling ground beetle species from eight within-city forest patches. In the second year, we also transferred ca. 800 tested individuals of two species to the laboratory to test for consistent behavioural differences (i.e. personality differences) under standardised conditions.

**Results:**

Individuals were generally more exploratory in more urbanized than in less urbanized areas but only in one year of the study. Exploratory behaviour was not predicted by population density but increased with temperature or showed a temperature optimum. Exploration was consistent over time and individuals that were more exploratory also took higher risks.

**Discussion:**

We demonstrated that species which are generally less directly exposed to human activities (e.g., most invertebrates) show behavioural responses to urbanization. Effects of urbanization were year-dependent, suggesting that other environmental conditions interacted with effects of urbanization on beetle behaviour. Furthermore, our results indicate that different personality compositions might cause behavioural differences among populations living in differently urbanized habitats.

## Introduction

Urbanization leads to substantial changes in natural habitats (McKinney, 2006) with profound effects on wildlife, from community level (e.g., Blair, 2001; McKinney, 2008; Rodewald et al., 2014) to within-species level. Within species, differences along rural–urban gradients have been detected in morphology (e.g., Weller & Ganzhorn, 2004; Giraudeau et al., 2014), physiology (e.g., Partecke, Schwabl & Gwinner, 2006), life history (e.g., Beck & Heinsohn, 2006; Rodewald & Shustack, 2008), and behaviour (e.g., Martin & Réale, 2008; Lehrer & Schooley, 2010; Sol, Lapiedra & Gonzalez-Lagos, 2013; Dahirel et al., 2017).

Behaviour is often the first response to altered conditions (Sih, Ferrari & Harris, 2011; Tuomainen & Candolin, 2011) with potentially large consequences on individuals (Sol, Lapiedra & Gonzalez-Lagos, 2013), population dynamics and biodiversity (Tuomainen & Candolin, 2011). Therefore, understanding individuals’ behavioural responses to urbanization is important for identifying which individuals or species may adapt to changing conditions or may disappear from altered habitats. Some behavioural traits, such as high exploration, high boldness and high aggressiveness, might be especially suited for coping with novel conditions (Sih, Ferrari & Harris, 2011; Phillips & Suarez, 2012; Lowry, Lill & Wong, 2013) and/or for colonizing urbanized habitats (Edelaar & Bolnick, 2012). Indeed, vertebrate urban populations are on average bolder (e.g., Prosser, Hudson & Thompson, 2006; Evans, Boudreau & Hyman, 2010; Uchida et al., 2016), less vigilant (Chapman, Rymer & Pillay, 2012), more aggressive (Evans, Boudreau & Hyman, 2010; Scales, Hyman & Hughes, 2011) and more exploratory (Martin & Réale, 2008) than their rural conspecifics.

In contrast to vertebrates, behavioural responses of invertebrates to urbanization are largely unknown. In general, work on urbanization is biased towards birds and mammals (Magle et al., 2012; Beninde, Veith & Hochkirch, 2015) and there was a call for considering other taxa (Beninde, Veith & Hochkirch, 2015), especially arthropods (McIntyre, 2000). Arthropods, and insects in particular, are important contributors to ecosystem functions (e.g., Mulder et al., 1999; Yang & Gratton, 2014; Griffiths et al., 2016). Thus, impacts of anthropogenic change on arthropods might have substantial consequences for whole ecosystems. In addition, some work suggests that birds and insects might respond differently to urbanization (Beninde, Veith & Hochkirch, 2015) which may be due to different ecological requirements and life histories. The vertebrate species (e.g., birds, squirrels, lizards) tested for behavioural differences across urbanization gradients are noticeable due to their size and/or behaviour, for instance to people walking in parks. Consequently, these species are likely not only indirectly (e.g., by habitat fragmentation or light pollution) but also directly (e.g., by supplemental feeding, harassing, unintentional disturbances by dogs or walking humans) influenced by human activities (following categorisation for (in)direct human effects by Clucas & Marzluff, 2011). In contrast, most invertebrate species (and some vertebrates) have a concealed lifestyle (e.g., they are small and/or hidden in vegetation or litter layer) which reduces direct contact with humans. As these less visible species should be mostly indirectly influenced by human activities, behavioural responses to urbanization might be different compared to openly visible mammals or birds. For instance, species with a concealed lifestyle may be influenced by urbanization if environmental conditions that change with urbanization (and that are not direct human interference) trigger behavioural responses.

Potential environmental drivers facilitating population differences in behaviour along urbanization gradients have been largely neglected. Abiotic drivers could include, amongst others, temperature, exposure to chemicals or humidity conditions. For instance, it is often warmer in more urbanized areas (Pickett et al., 2001) and temperature influences behaviour, particularly of ectothermic organisms. Biotic drivers may include population density and intraspecific competition, altered prey items, pathogens or unfamiliar predators. For example, urbanization often influences abundance (e.g., Shochat et al., 2006). Population density, in turn, affects behaviours such as exploration-activity (Le Galliard, Paquet & Mugabo, 2015), aggression (Cubaynes et al., 2014), sociability (Le Galliard, Paquet & Mugabo, 2015), foraging (Mobæk et al., 2012) and dispersal (Tuda & Shima, 2002).

Here, we studied effects of urbanization on ground beetle behaviour. Ground beetles are suitable invertebrates for urbanization studies as they depend heavily on environmental factors, such as temperature, humidity or food availability (Lövei & Sunderland, 1996; Rainio & Niemelä, 2003). Consequently, they are known to react quickly to environmental change, making behavioural responses to urbanization likely. Urbanization has already been shown to affect carabids, such as their species composition and abundance (Weller & Ganzhorn, 2004; Sadler et al., 2006; Niemelä & Kotze, 2009; Magura, Lovei & Tothmeresz, 2010; Davies, Bennie & Gaston, 2012; Kotze et al., 2012).

We expected more exploratory and high-risk taking ground beetle individuals to cope better with new challenges and new niches and/or to be more prone to colonize urbanized areas. We predicted this to result in a higher proportion of consistently more exploratory or risk-prone individuals in urbanized compared to rural areas, leading to increased population averages in these behaviours in more urbanized areas. Consistent individual differences in behaviour (’animal personality differences’ sensu Dall, Houston & McNamara, 2004) have been recorded in many species (Gosling, 2001), recently also including invertebrate taxa (Kralj-Fišer & Schuett, 2014).

In a two-year field study we tested ground beetles from differently urbanized forests for differences in their exploratory and risk-taking behaviour. We considered temperature and population density as potential environmental drivers facilitating behavioural differentiation between populations living under different levels of urbanization. We studied individuals of four forest-dwelling ground beetle species from eight within-city forest patches of two different urbanization levels (four populations per level). The four ground beetle species differ in their dispersal abilities and niche widths (cf. Lindroth, 1985/6; Turin, 2000). In the second year, we additionally tested individuals of two species repeatedly for their exploratory and risk-taking behaviour in the laboratory to assess consistent personality differences.

## Material & Methods

### Study species and study sites

We studied four ground beetle species that are all predators but that differ in other traits. Two species, *Abax parallelepipedus* (Piller & Mitterpacher, 1783) and *Pterostichus oblongopunctatus* (F., 1787), are stenotopic, only occurring in woodlands (Lindroth, 1985/6); two species, *Carabus nemoralis* Müller, 1764, and *Nebria brevicollis* (F., 1792), are eurytopic and can also be found in more open habitats, such as gardens or parks (Lindroth, 1985/6). The selected species also vary in their flight ability and hence dispersal: *N. brevicollis* and *P. oblongopunctatus* are macropterous and wing-polymorphic, respectively (i.e., at least some individuals might be able to fly), whereas *A. parallelepipedus* and *C. nemoralis* cannot fly as they have reduced alae (cf Homburg et al., 2014). Reproductive seasons, in which adults show epigaeic activity to search for e.g., food or mating partners, are in March–June (*C. nemoralis*), April–June (*P. oblongopunctatus*), April–August (*A. parallelepipedus*), and August–September (*N. brevicollis*) (Turin, 2000). Our study was conducted during the reproductive season of the studied species, except for *N. brevicollis*, for which also tenerals (freshly hatched beetles) occurred (see ‘Trapping’).

In the city of Hamburg, Germany, eight forest sites of different levels of urbanization (four ‘low’ *vs.* four ‘high’) were studied. Forests were typical mixed deciduous forests dominated by either beech, *Fagus sylvatica*, oak, *Quercus robur*, or other native tree species. We obtained an urbanization score for each site following the method developed by Czúni, Lipovits & Seress (2012) and validated by Seress et al. (2014). This method calculates an urbanization score of 1 km^2^ around the site using aerial images from GoogleMaps^®^ based on the predominant landscape category (buildings, paved roads, vegetation) in each of 100 equally sized squares. The lowest four urbanization scores were assigned ‘low’, the highest four scores ‘high’ urbanization level (Table S1). As the surrounding is taken for this classification, forests that were less urbanized were larger than more urbanized forests (Table 1; Table S1). Distances of our trapping sites to the respective forest edges did not differ between sites of low and high urbanization (Table 1). Field work was conducted under license from the respective German authority (Behörde für Umwelt und Energie der Freien und Hansestadt Hamburg, Amt für Naturschutz, Grünplanung und Energie; Az.: 897.00-02.6).

**Table 1:** Comparison of environmental variables and density proxies of the studied species between sites of low and high level of urbanization. Given is the mean (±SE) of the respective variables. Variables were either compared with general linear models (GLMs) or with linear mixed effects models (LMMs) with normal error structures. For LMMs, ‘forest site’ was taken as random effect. Temperature data are based on temperature measures during behavioural tests (one value per tested beetle). Bold *p*-values denote significance.

			Comparison				
	UL: low (*N* = 4)	UL: high (*N* = 4)	Test	*X*	Transf.	Test-statistic	*P*
Forest size [ha]	284.25 ± 163.78	15.25 ± 6.14	GLM	UL	log	*F*_1,6_ = 18.55	**0.005**
Distance to forest edge [m]	129.75 ± 29.34	69.50 ± 12.86	GLM	UL	log	*F*_1,6_ = 3.25	0.122
Temperature [°C]							
2015: *N* = 2, 189	15.1 ± 0.1	15.9 ± 0.1	LMM	UL	none	${X}_{1}^{2}=0.56$	0.454
2016: *N* = 767	16.5 ± 0.3	17.6 ± 0.3		Year		${X}_{1}^{2}=96.16$	**<0.001**
Individuals trapped [mean 10 days^−1^ trap^−1^]							
AP (2015)	0.029 ± 0.02	0.226 ± 0.13	GLM	UL	sqrt	*F*_1,6_ = 2.52	0.164
CN (2015)	0.020 ± 0.01	0.299 ± 0.11	GLM	UL	sqrt	*F*_1,6_ = 9.11	**0.023**
NB (2015)	2.126 ± 0.78	1.372 ± 0.66	LMM	UL	sqrt	${X}_{1}^{2}=0.03$	0.854
NB (2016)	6.075 ± 4.12	4.012 ± 1.86		Year		${X}_{1}^{2}=2.94$	0.086
PO (2015)	2.179 ± 0.69	1.160 ± 0.47	LMM	UL	sqrt	${X}_{1}^{2}=1.62$	0.204
PO (2016)	1.923 ± 0.56	1.398 ± 0.47		Year		${X}_{1}^{2}=0.01$	0.932

**Notes.**

AP *Abax parallelepipedus* CN *Carabus nemoralis* NB *Nebria brevicollis* PO *Pterostichus oblongopunctatus* transf. transformation of response (sqrt, square-root taken; log, natural logarithm taken) UL urbanization level (‘low’ *vs.* ‘high’) X explanatory variable or fixed effect

### Trapping

Trapping was conducted between 20 April–11 June 2015 and 26 April–7 June 2016. At each site we installed live pitfall traps with ca. 10 m between traps (2015: 25 traps in a 5 × 5 grid; 2016: 15 traps in a 5 × 3 grid). Modified pitfall traps were used to reduce predation of small carabids by larger beetles: traps consisted of two plastic cups (10 cm diameter) of different height (5 cm *vs.* 10 cm). The smaller cup was placed inside the larger cup, such that the opening of both cups was at the same height, creating two levels in the trap. Holes (ca. 5 mm) at the bottom of the smaller inner cup allowed smaller beetles to escape into the lower level but were too small for larger beetles to follow. Small holes in the bottom of the outer cup served as drain. Both, inner and outer cup of each trap were baited with red wine on a piece of cellulose (Marcus et al., 2015). Pitfall traps were dug in the ground and covered with metal mesh. Traps were controlled once per week per site. Trapped beetles were transferred into plastic vials (50 ml, ca. 4.5 cm diameter), identified to species level and sexed (the tarsi of the front legs of males are wider than those of females, Forsythe, 2000).

From pitfall trapping in the same forest patches in earlier years and parallel to this study the carabid fauna is well known so that misidentifications due to the occurrence of similar species can be ruled out. In only one site we detected a species (*Nebria salina* Fairmaire and Labourbne, 1854) that is difficult to distinguish from *N. brevicollis* in the field. Therefore, we excluded any *N. brevicollis* that we had trapped at this site from our analysis (*N* = 6; Table S2).

### Behavioural tests

Trapped beetles were individually tested in the field for their behaviour. In 2015 we tested individuals of all four species; in 2016 we tested *N. brevicollis* and *P. oblongopunctatus*. First, we measured individuals’ activity in a novel environment test, also referred to as ‘open field’ test (Réale et al., 2007), a test that is often applied to classify exploratory behaviour (Jones & Godin, 2010; Lantová et al., 2010; Schuett, Laaksonen & Laaksonen, 2012). Directly after, we tested individuals for their risk-taking behaviour (thanatosis; see Supplemental Information 1). Since only 21% of individuals showed thanatosis behaviour (see Supplemental Information 1), we did not test for relationships between risk-taking behaviour and urbanization or other environmental variables.

The novel environment (open white plastic box: 25 × 36.5 × 10 cm) was divided into 28 squares. At the beginning of a test, a randomly chosen beetle was placed in one specific field in the inner area of the novel environment. Subsequently, the number of square visits within 90 s of test begin was noted as measure of exploratory behaviour. In 2015 we released the individuals in vicinity of the trapping grid after behavioural testing. Multiple testing of the same individual within that year could be avoided since captured beetles were colour-marked (edding^®^ 751, edding, Wunstorf, Germany) on their elytra. In 2016 we transferred each individual after behavioural testing into a numbered plastic vial (50 ml, 4.5 cm diameter) for further behavioural tests under standardized laboratory conditions (see ‘Behavioural tests in laboratory 2016’).

### Environmental variables: population density and temperature

We used the total number of individuals caught per species for each site and each year as a proxy for carabid population size (and density, Baars, 1979). Beetles were caught in extra pitfall traps that had been set at each site throughout the study periods (*N* = 4 per site; roughly 50 m away from our grid). We calculated the number of individuals per trap and 10 trapping days and species (variable: density) from the total catches.

We measured the temperature during behavioural testing at each forest site using data loggers (Voltcraft DL-121TH, Hirschau, Germany) every 10 min. Temperature during the tests did not differ between sites of low and high level of urbanization (Table 1). However, in 2016 it was warmer than in 2015 (Table 1).

### Behavioural tests in laboratory 2016

To test for consistent personality differences, we twice retested all individuals in the laboratory under standardized conditions (16 L : 8 D illumination cycle with full spectrum fluorescent light; 17.9 °C : 7.3 °C temperature cycle; 60% : 80% humidity) for their exploratory and risk-taking behaviour. To minimize potential effects of different conditions experienced in the field and in the laboratory on behavioural responses, we handled and tested the beetles in the same way in the laboratory as in the field. Temperature conditions in the laboratory corresponded to long-term temperature minimum and maximum means in May (Deutscher Wetterdienst; station: Fuhlsbüttel, Hamburg). Individuals were tested in random order on day 2 and day 7 (field test = day 0). After the first behavioural test series in the laboratory individuals were fed with one *Calliphora* sp. pupa each.

### Statistical analyses

We analyzed the effect of urbanization (‘low’ *vs.* ‘high’) on exploratory behaviour (response variable: number of square visits) of each study species separately using linear mixed effects models (LMMs). Fixed effects included urbanization level, density as well as sex of the tested individual. Temperature during testing was added as covariate (temperature and temperature^2^ to test for a possible temperature optimum curve). Furthermore, we added an interaction between density and sex as both sexes may show different density-dependent effects on behaviour. To test whether the effect of urbanization on behaviour depended on environmental conditions in different years, we also added the interaction between year and urbanization level for those species (*N. brevicollis*, *P. oblongopunctatus*) that were studied in 2015 and in 2016. The variable year thus captures all changes in abiotic and biotic conditions, such as humidity or food availability, that are not included in the variables temperature and density. Forest site, observer and week of testing were added as random terms allowing for random intercepts. If required (see Table 2; Table S3), the number of square visits was square-root transformed to meet model assumptions. We did not use Poisson error structure since those models were overdispersed. Models were manually simplified step-wise (Crawley, 2002), by taking each term out in turn and comparing the models without each term against the more complex model using likelihood ratio tests (Crawley, 2007). The highest non-significant term at each step was removed given its removal did not significantly reduce the power of the model (as indicated by the likelihood ratio tests).

**Table 2:** Summary of test statistics from LMMs with the number of square visits in a novel environment as response. Coefficients (coeff.) in brackets: coefficients of non-significant terms just before dropping the terms; other coefficients (not in brackets): from minimal adequate model (please note coefficients in brackets cannot be compared to coefficients from the minimal adequate models, since the simplification alters coefficients); bold *p*-values denote significant effects; coefficients for a factor level (specified in square brackets) give the difference to the reference level.

Species (Year)	Random term	Variance	Fixed effect	Coeff.^a^	*X*^2^	*df*	*P*	Transf.	*N*
AP	Observer	0	(Mean)	28.88				none	239
(2015)	Site	115.92	Density : Sex [female]	(−10.33)	0.44	1	0.5064		
	Week	57.94	Density	(−5.69)	0.22	1	0.6377		
	(Residual)	831.88	Sex [female]	−7.68	3.97	1	**0.0463**		
			Temperature^2^	(−0.22)	1.76	1	0.1847		
			Temperature	1.58	5.48	1	**0.0193**		
			UL [low]	(−14.57)	2.91	1	0.0878		
CN	Observer	0.19	(Mean)	2.42				sqrt	321
(2015)	Site	0	Density : Sex [female]	(2.09)	3.04	1	0.0813		
	Week	0.08	Density	(−0.65)	0.69	1	0.4064		
	(Residual)	2.88	Sex [female]	−0.63	8.06	1	**0.0045**		
			Temperature^2^	(<−0.01)	<0.01	1	0.9755		
			Temperature	0.07	5.91	1	**0.0150**		
			UL [low]	−0.57	4.41	1	**0.0358**		
NB	Observer	6.61	(Mean)	−56.68				none	864
(2015 +	Site	16.11	Density : Sex [female]	(−0.56)	0.49	1	0.4824		
2016)	Week	70.95	UL [low] : Year [2016]	11.67	9.46	1	**0.0021**		
	(Residual)	460.22	Density	(−0.65)	1.37	1	0.2419		
			Sex [female]	(1.47)	0.95	1	0.3298		
			Temperature^2^	−0.31	25.48	1	**<0.0001**		
			Temperature	11.07					
			UL [low]	−7.12					
			Year [2016]	0.89					
PO	Observer	0.04	(Mean)	2.07				sqrt	1,532
(2015 +	Site	0.16	Density : Sex [female]	(0.01)	0.02	1	0.8773		
2016)	Week	0.04	UL [low] : Year [2016]	0.58	7.49	1	**0.0062**		
	(Residual)	3.18	Density	(−0.09)	1.12	1	0.2892		
			Sex [female]	−0.56	36.37	1	**<0.0001**		
			Temperature^2^	−0.01	5.79	1	**0.0161**		
			Temperature	0.26					
			UL [low]	−0.35					
			Year [2016]	−1.23					

**Notes.**

AP *Abax parallelepipedus* CN *Carabus nemoralis* NB *Nebria brevicollis* PO *Pterostichus oblongopunctatus* transf. transformation of response (sqrt square-root taken) UL urbanization level (‘low’ vs. ‘high’)

a Please note that coefficients are not back-transformed for those analyses in which response was transformed.

Behavioural consistency of the number of square visits within (lab-lab) and between situations (field-lab) was assessed in two ways. First, we estimated repeatabilities from LMMs with ID as random term. When field data were involved, ambient temperature (temperature and temperature^2^) was included as fixed term and adjusted repeatabilities assessed. As before, we square-root transformed our response variable, if needed, to meet model assumptions. Second, we used Spearman rank correlations to assess rank consistency of individuals in their number of square visits along the test series.

All statistics were carried out in R (R Core Team, 2016). LMMs were conducted using the lme4 package (Bates et al., 2015), repeatabilities and their confidence intervals were estimated using the rptR package (version 0.9.1, Schielzeth, Stoffel & Nakagawa, 2017), and Spearman rank correlations and their confidence intervals were computed using the RVAideMemoire package (version 0.9-64, Hervé, 2016). Graphs are based on raw data.

## Results

### Trapping and behavioural testing in the field

All species were trapped in all eight sites apart from *A. parallelepipedus* which was trapped in six sites only (Table S2). In 2015 we tested 2189 individuals (44% females; Table S2) at a mean temperature of 15.5 °C (±0.1 SE); in 2016 we tested 767 individuals (53% females; Table S2) at a mean temperature of 17.0 °C (±0.2 SE). In total, we tested 239 *A. parallelepipedus* (2015), 321 *C. nemoralis* (2015), 864 *N. brevicollis* (2015 and 2016) and 1,532 *P. oblongopunctatus* (2015 and 2016). Population densities per year and species did not differ between forests of different urbanization, except for *C. nemoralis* which was trapped more frequently in highly urbanized sites (Table 1).

### Predictors of behaviour in the field

**Figure 1:**
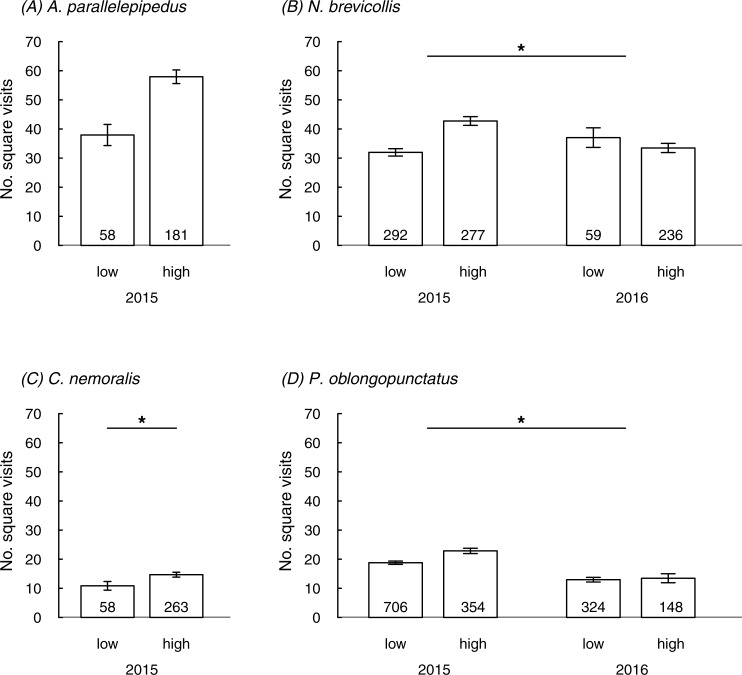
The number of square visits (mean ± SE) in a novel environment shown by individuals from differently urbanized forests (‘low’ vs. ‘high’) of (A–D) four carabid species in field tests. Numbers in bars indicate sample sizes. Asterisks in (B) and (D) denote significant interaction, in (C) indicate significant difference.

In three species the number of square visits in the field was linked to the urbanization level or the interaction between urbanization and year; a non-significant trend for an effect of urbanization was also found in the fourth species (*A. parallelepipedus*) (Table 2; Fig. 1). In line with prediction, individuals mostly showed more square visits in more urbanized than in less urbanized sites: this was generally true in 2015 but in 2016 patterns were less clear (significant interaction between urbanization and year in *N. brevicollis* and *P. oblongopunctatus*). The number of square visits was temperature-dependent for all species (Table 2) and either increased with rising temperatures (*A. parallelepipedus* and *C. nemoralis*) or showed a temperature optimum (*N. brevicollis* and *P. oblongopunctatus*). Sexes differed in their behaviour: in all species, except for *N. brevicollis*, females had less square visits than males (Table 2). Population densities did not predict the number of square visits in any species (Table 2).

### Behavioural consistency

The number of square visits in the field (on day 0) and in the laboratory test (on day 2) was rank-consistent for individuals of both species, *N. brevicollis* (*N* = 295 individuals) and *P.* *oblongopunctatus* (*N* = 472 individuals), but only repeatable in *N. brevicollis* (Table S4). In the laboratory, the number of square visits was moderately rank-consistent (0.260–0.307) and repeatable (0.249–0.321) over time in both species no matter whether sexes were analyzed separately or pooled (Table S4). Individuals that originated from highly urbanized sites were also rank-consistent and repeatable in the number of square visits in the laboratory in both species; individuals from less urbanized areas were only rank-consistent and repeatable for *P. oblongopunctatus* but not for *N. brevicollis* (Table S4).

## Discussion

In line with prediction, ground beetles were more exploratory (i.e., showed more square visits) in highly urbanized forests compared to less urbanized forests (three out of four species) but for those two species studied over two years, effects were year-dependent. Exploratory behaviour was not influenced by population density but temperature-dependent in all and sex-dependent in three species. Individuals consistently differed in their exploratory behaviour over time, i.e., personality differences existed among individuals (tested in two species). Individuals were generally consistent in their exploratory behaviour across tests in the field and in the laboratory, suggesting behaviour tests in the laboratory can be used to predict behaviour when tested in the field. Exploratory individuals were also bolder (less likely to show thanatosis; Supplemental Information) in three species.

Our results that ground beetles vary in their behaviour between differently urbanized areas (at least under certain conditions) are in line with the few other existing studies that tested effects of anthropogenic change on invertebrate behaviour. Insects varied in their behaviour as response to traffic noise (Lampe et al., 2012; Lampe, Reinhold & Schmoll, 2014; Orci, Petroczki & Barta, 2016) or reacted differently towards chemicals when originating from urbanized as compared to rural sites (Tuzun et al., 2015). However, there are still too few studies to draw general conclusions about behavioural responses of invertebrates to urbanization. It is possible that such responses are generally less pronounced in invertebrates and therefore less often published compared to responses in vertebrates. Moreover, year-dependent effects of urbanization on behaviour—as revealed in our study—point towards more complex patterns where other environmental factors interact with urbanization. For a deeper understanding of behavioural responses of invertebrates to urbanization more long-term studies are necessary. Nevertheless, results reported here were quite similar across species even though our study species differed in their niche width, dispersal ability and reproductive season. This indicates that the patterns we found might be more general, potentially even covering many other forest-dwelling arthropods.

The factors that could contribute to behavioural differences in less and highly urbanized areas are manifold (e.g., Tuomainen & Candolin, 2011). We here assessed the possibilities that differences in temperature or population densities could lead to behavioural differences in differently urbanized areas. While we reported temperature effects on behaviour, temperature during tests did not differ between less and highly urbanized forests. Contrary to our findings, temperatures are often higher in urban than rural areas (Pickett et al., 2001) but this might be less pronounced in city forest patches of different urbanization or during certain times of the year. Furthermore, in only one species the population density was higher in highly urbanized areas and we generally detected no effect of density on behaviour. Other factors, that could explain differences in behaviour of ground beetles between the differently urbanized sites may, for instance, include litter layer depth (Marcus et al., 2015) or food availability. The litter layer is the primary habitat for ground beetles and their prey; and litter decomposition rate increases along urbanization gradients (Pouyat, McDonnell & Pickett, 1997), resulting in reduced litter depth (Van Nuland & Whitlow, 2014). Moreover, activity of ground beetles is reduced with higher food supply (Lenski, 1984). Thus, if reduced litter layers (or other environmental factors) in more urbanized areas lead to reduced availability of prey, increased locomotory activity and exploratory behaviour could be beneficial for the ground beetles for locating food. Exploratory or bold behaviour might also be generally favourable in these forests: urbanization often leads to changes in the structure of (invertebrate) assemblages, including prey items of ground beetles (Steinberg et al., 1997; Van Nuland & Whitlow, 2014; Bogyo et al., 2015), such that beetles may encounter novel prey items. Indeed, abundance of non-native earthworms increased with urbanization (Steinberg et al., 1997), with earthworms being common prey items of carabids (cf Turin, 2000).

Year-dependent effects of urbanization on exploratory behaviour suggest that other environmental conditions, which varied between years, interacted with the effect of urbanization on beetle behaviour. Our results are corroborated by one of the few studies that tested temporal effects of urbanization: arthropod biodiversity changed with urbanization but differently through time (Van Nuland & Whitlow, 2014). Environmental conditions that interact with effects of urbanization could be changes in food availability, soil moisture or depth of the litter layer. If, for instance, litter layers had been thicker in 2016, this could have led to a reduced effect of high urbanization on behaviour (e.g., because prey may be more abundant in thicker layers, potentially reducing the need to explore). Future studies should consider potential environmental variables that might interact with effects of urbanization on studied traits. For testing such interactions, more long-term studies are urgently needed.

Exploration was temperature-dependent. While this result is not surprising given that poikilotherms crucially depend on surrounding temperatures, it highlights how important it is to consider ambient temperature in behavioural and urbanization studies. This is particularly true for field studies, where temperature conditions are not standardized. Temperatures are often higher in urban than rural areas (Pickett et al., 2001). Given that the investigated beetles reacted to increased temperature with more exploratory behaviour, it is possible that different temperatures across the differently urbanized forests caused different behavioural levels among populations. Yet at least during behavioural testing we did not find temperature differences between less and more urbanized forests. Nevertheless, future studies should further investigate whether temperature differences between urban and rural areas can drive behavioural responses to urbanization in poikilotherms.

Reported behavioural differences at population level could arise if individuals (a) react flexibly to altered environmental conditions and/or (b) if individuals consistently differ in their behaviour (i.e., show animal personality differences, sensu Dall, Houston & McNamara, 2004) (Sih, Ferrari & Harris, 2011; Brown, 2012; Lowry, Lill & Wong, 2013; Sol, Lapiedra & Gonzalez-Lagos, 2013). In our study we found consistent personality differences in exploratory behaviour in the two species that were also tested in the laboratory (*N. brevicollis* and *P. oblongopunctatus*). Even though we cannot conclusively differentiate between the above mechanisms (and both mechanisms might work at the same time), our data provide some valuable first insights. Behaviour was repeatable and/or (rank-) consistent for at least a few days after individuals had been taken out of their natural environment and kept under standardized conditions in the laboratory. These results may suggest that personality compositions varied between differently urbanized areas, or that individuals reacted differently to the environments (I × E), or that influences of environmental conditions on behaviour still carried-over when conditions changed. However, in the latter case we would not expect to find repeatable behavioural differences among individuals within populations in sites of similar urbanization (assuming homogenous environmental conditions). Rather, individuals should be all similar in their response, leading to low repeatability, despite high individual consistency. Tentatively, our results seem to suggest that different personality types are adapted to different environmental conditions with consistently more exploratory individuals being favoured in highly urbanized sites. Such behavioural changes in urbanized areas might lead to altered population dynamics and potentially even modified community structure. For instance, more exploratory individuals are often also higher risk takers (e.g., Van Oers et al., 2004; this study), more aggressive (e.g., Verbeek, Boon & Drent, 1996; Schuett, Dall & Royle, 2011) and may often follow different life-history strategies than less exploratory individuals (Réale et al., 2010); this likely influences population dynamics.

We showed that ground beetles exhibit consistent personality differences in both, the reproductive (*P. oblongopunctatus*) and non-reproductive season (*N. brevicollis*). Males and females of both species showed personality differences in exploratory behaviour and higher exploration was associated with higher risk-taking in both sexes for three species. In contrast, sexes differed in their mean levels of exploration: with the exception of *N. brevicollis*, males of all species were more exploratory than females. This may suggest that sexes vary in their reaction towards urbanization. In addition, all species, except *N. brevicollis*, were measured during their reproductive season. Thus, males may generally be more exploratory and/or active during the reproductive phase in order to locate mates for reproduction. Higher trapping rates of males compared to females during the reproductive phase support this idea (Drees & Huk, 2000; Weber & Heimbach, 2001).

### Conclusions

Similar to mammals and birds, which are often directly influenced by human disturbances in city-forests, we showed that also invertebrate species seem to be more exploratory in highly urbanized compared to less urbanized areas but that effects of urbanization depended on other environmental variables. More long-term studies are required to identify such environmental variables. Furthermore, common garden and/or translocation experiments are now needed to shed further light into the mechanisms underlying population differences in behaviour across differently anthropogenically altered environments.

##  Supplemental Information

10.7717/peerj.4360/supp-1 Supplemental Information 1 Supplemental Methods and Results: Thanatosis

10.7717/peerj.4360/supp-2 Table S1 Characteristics of the eight study sites

10.7717/peerj.4360/supp-3 Table S2 Summary of population density, trapping and behavioural testing per species and site

10.7717/peerj.4360/supp-4 Table S3 Summary of test statistics from LMMs assessing links between the number of square visits in a novel environment (response) and the occurrence of thanatosis in both field and laboratory tests In the field each individual was tested once; in the laboratory each individual was tested twice. Coefficients (coeff.) in brackets: coefficients of non-significant terms just before dropping the terms, other coefficients (not in brackets): from minimal adequate model (note coefficients in brackets cannot be compared to coefficients from the minimal adequate models, since the simplification alters coefficients); bold p-value denotes significant effect; coefficients for factor levels give the difference to the reference level.

10.7717/peerj.4360/supp-5 Table S4 Summary of behavioural consistencies for the number of square visits from (adjusted) repeatability (*r*) or Spearman rank-correlations (*R*_*s*_) for two different species and data subsets in 2016 For data including field tests adjusted repeatabilities were calculated with ambient temperature (temperature and temperature^2^) as fixed term. Bold test statistics denote significance.

10.7717/peerj.4360/supp-6 Figure S1 The number of square visits (mean ± SE) in a novel environment for individuals that did or did not show thanatosis in (A–D) both field and (E–F) laboratory tests In the field each individual was tested once; in the laboratory each individual was tested twice. Numbers in bars indicates number of trials (in field: number of trials = number of individuals). Asterisks indicate significant differences.

10.7717/peerj.4360/supp-7 Data S1 Raw data

## Additional Information and Declarations

### Field Study Permissions

The following information was supplied relating to field study approvals (i.e., approving body and any reference numbers): Fieldwork was conducted under a license from the respective German authority (Behörde für Umwelt und Energie der Freien und Hansestadt Hamburg, Amt für Naturschutz, Grünplanung und Energie; Az.: 897.00-02.6).

### Data Availability

The following information was supplied regarding data availability: The raw data is provided as a Supplemental File.
