# Supplementary material for: Ground beetles in city forests: does urbanization predict a personality trait?
Source: PeerJ. 2018 Feb 20;6:e4360. doi: 10.7717/peerj.4360 (PMC5824674; doi:10.7717/peerj.4360)
Supplement: Supplemental Information 1 [file peerj-06-4360-s001.docx]

# Supplemental Information

**Ground beetles in city forests: Does urbanization predict a personality trait?**

Wiebke Schuett^1^, Berit Delfs^1^, Richard Haller^1^, Sarah Kruber^1^, Simone Roolfs^1^, Desiree Timm^1^, Magdalena Willmann^1^, Claudia Drees^1^

^1^Zoological Institute, Biocenter Grindel, Universität Hamburg, Hamburg, Germany

# Methods

Directly after the novel environment test we tested the beetles for their death-feigning behaviour, also labelled ‘thanatosis’ (Bleich, 1927) or ‘tonic immobility’ (Rogers & Simpson, 2014). Death-feigning behaviour reflects antipredator behaviour shown in the wild (Edelaar et al., 2012); and flour beetles, *Tribolium castaneum*, selected for a lower frequency and shorter duration of thanatosis had a lower survival rate under predation risk than those selected for a higher thanatosis frequency (Nakayama & Miyatake, 2010). Therefore, we used thanatosis behaviour as a proxy for risk-taking.

The beetle was turned on its back with insect forceps (simulating a predation event) and placed again in the novel environment arena. We measured whether or not the beetle showed thanatosis within 90 s (including the latency until thanatosis). The beetle showed thanatosis if it completely stopped any movements. Alternatively, the beetle did not show thanatosis if it either continuously moved its legs for 90 s or turned itself onto its feet during the test. When thanatosis occurred we measured its duration for up to 90 s.

We tested for an association between the novel environment and thanatosis behaviour with a LMM for each species: the models included the number of square visits as response, the occurrence of thanatosis (binary variable), sex and the interaction between sex and occurrence of thanatosis as fixed effects. Random terms in all models were forest site, observer and week of testing.

We did not statistically test for links between urbanization and thanatosis for two reasons. First, only few individuals showed thanatosis, especially in the laboratory (Table S2). Second, the behaviour in the novel environment could have influenced subsequent thanatosis because the novel environment and thanatosis tests were conducted directly after one another.

# Results

In the field, only 21% (*N* = 634) of the individuals (*N* = 2956) showed thanatosis (Table S2). Thanatosis occurred most frequently within 5 s after the test start (*N* = 427) and lasted on average 56.82 s (± 1.34 SE; *N* = 634). Most individuals that did not show thanatosis within 90 s (*N* = 2322) were able to turn themselves from the back onto their feet (*N* = 2147). The more square visits an individual had, the less likely it was to show thanatosis in all species except *C. nemoralis* (Table S3; Fig. S1 A-D). The link between thanatosis and the number of square visits was independent of sex in all four species (Table S3).

In the laboratory, only 3% on day 2 (*N* = 24) and 4% on day 7 (*N* = 28) of the individuals (*N* = 767) showed thanatosis. Again, individuals that showed no thanatosis had more square visits in the laboratory for both species tested (Table S3; Fig. S1 E-F).

# References

Bleich, O. E. (1927). Thanatose und Hypnose bei Coleopteren. Experimentelle Untersuchungen. *Zeitschrift für Morphologie und Ökologie der Tiere, 10*, 1-61.

Edelaar, P., Serrano, D., Carrete, M., Blas, J., Potti, J., & Luis Tella, J. (2012). Tonic immobility is a measure of boldness toward predators: an application of Bayesian structural equation modeling. *Behavioral Ecology, 23*(3), 619-626. doi: 10.1093/beheco/ars006

Nakayama, S., & Miyatake, T. (2010). Genetic trade-off between abilities to avoid attack and to mate: a cost of tonic immobility. *Biology Letters, 6*(1), 18-20. doi: 10.1098/rsbl.2009.0494

Rogers, S. M., & Simpson, S. J. (2014). Thanatosis. *Current Biology, 24*(21), R1031-R1033. doi: 10.1016/j.cub.2014.08.051
