# Supplementary material for: Ground beetles in city forests: does urbanization predict a personality trait?
Source: PeerJ. 2018 Feb 20;6:e4360. doi: 10.7717/peerj.4360 (PMC5824674; doi:10.7717/peerj.4360)
Supplement: Table S1 [file peerj-06-4360-s002.docx]

| Site | Year | UL | Size [ha] | Distance forest  edge [m] | Geographic coordinates |
| --- | --- | --- | --- | --- | --- |
| Bornmoor | 2015 | High | 13 | 88 | 53.591217° N, 9.895903° E |
| Bornmoor | 2016 |  |  | 84 | 53.591217° N, 9.889694° E |
| Borsteler Jäger | 2015 + 2016 | High | 10 | 58 | 53.618122° N, 9.986786° E |
| Fischbeker Heide | 2015 + 2016 | Low | 773 | 116 | 53.437655° N, 9.877291° E |
| Marienhöhe | 2015 + 2016 | Low | 74 | 200 | 53.573945° N, 9.788399° E |
| Meyers Park | 2015 + 2016 | High | 33 | 95 | 53.466200° N, 9.943583° E |
| Niendorfer Gehege | 2015 + 2016 | Low | 142 | 144 | 53.614976° N, 9.943941° E |
| Ohmoor | 2015 + 2016 | High | 5 | 39 | 53.641575° N, 9.963214° E |
| Stadtpark | 2015 + 2016 | Low | 148 | 59 | 53.595464° N, 10.012349° E |

Size, size of forest; UL, urbanization level.
