# Supplementary material for: Ground beetles in city forests: does urbanization predict a personality trait?
Source: PeerJ. 2018 Feb 20;6:e4360. doi: 10.7717/peerj.4360 (PMC5824674; doi:10.7717/peerj.4360)
Supplement: Table S2 [file peerj-06-4360-s003.docx]

**Table S2** Summary of population density, trapping and behavioural testing per species and site.

| Species | Site | Density ^a^ | No. ID trapped once ^b^ | No. recaptures | No. ID tested ^c^ | | Mean no. square visits | | SE no. square visits | | % thanatosis | |
| --- | --- | --- | --- | --- | --- | --- | --- | --- | --- | --- | --- | --- |
|  |  |  |  |  | males | females | males | females | males | females | males | females |
| AP (2015) | Bornmoor | 0.12 | 16 | 3 | 11 | 5 | 45.00 | 31.00 | 12.57 | 15.24 | 27 | 0 |
|  | Borsteler Jäger | 0.60 | 109 | 18 | 50 | 44 | 59.32 | 49.68 | 4.82 | 3.92 | 34 | 45 |
|  | Fischbeker Heide | 0.00 | 3 | 0 | 2 | 1 | 51.00 | 42.00 | 8.00 | - | 0 | 0 |
|  | Marienhöhe | 0.10 | 47 | 25 | 23 | 14 | 42.78 | 26.00 | 6.19 | 6.86 | 4 | 36 |
|  | Meyers Park | 0.19 | 81 | 10 | 46 | 25 | 67.02 | 64.00 | 3.90 | 6.56 | 11 | 20 |
|  | Niendorfer Gehege | 0.01 | 18 | 0 | 8 | 10 | 36.00 | 42.00 | 9.51 | 8.93 | 63 | 20 |
|  | Ohmoor | 0.00 | 0 | 0 | 0 | 0 | - | - | - | - | - | - |
|  | Stadtpark | 0.00 | 0 | 0 | 0 | 0 | - | - | - | - | - | - |
| CN (2015) | Bornmoor | 0.57 | 137 | 15 | 122 | 14 | 13.54 | 12.00 | 1.04 | 2.67 | 20 | 36 |
|  | Borsteler Jäger | 0.25 | 62 | 14 | 28 | 30 | 20.71 | 12.00 | 2.40 | 2.62 | 18 | 60 |
|  | Fischbeker Heide | 0.00 | 1 | 0 | 1 | 0 | 5.00 | - | - | - | 0 | - |
|  | Marienhöhe | 0.03 | 52 | 8 | 34 | 13 | 12.47 | 5.46 | 2.15 | 1.55 | 44 | 23 |
|  | Meyers Park | 0.03 | 5 | 0 | 3 | 2 | 6.33 | 14.00 | 3.53 | 13.00 | 0 | 0 |
|  | Niendorfer Gehege | 0.03 | 2 | 0 | 1 | 1 | 26.00 | 1.00 | - | - | 100 | 100 |
|  | Ohmoor | 0.34 | 91 | 3 | 43 | 21 | 17.65 | 14.14 | 2.66 | 3.25 | 51 | 48 |
|  | Stadtpark | 0.02 | 9 | 1 | 4 | 4 | 22.25 | 3.50 | 4.01 | 1.50 | 0 | 50 |
| NB (2015) | Bornmoor | 0.44 | 16 | 1 | 10 | 6 | 49.90 | 61.50 | 13.03 | 10.62 | 0 | 0 |
|  | Borsteler Jäger | 3.17 | 214 | 4 | 60 | 58 | 38.28 | 40.78 | 3.16 | 2.73 | 12 | 3 |
|  | Fischbeker Heide | 0.00 | (3) ^d^ | 0 | ‑ | ‑ | ‑ | ‑ | - | ‑ | ‑ | ‑ |
|  | Marienhöhe | 3.35 | 1026 | 30 | 79 | 61 | 24.63 | 34.46 | 2.17 | 3.37 | 27 | 18 |
|  | Meyers Park | 0.32 | 25 | 0 | 13 | 12 | 34.23 | 35.33 | 6.62 | 7.34 | 31 | 25 |
|  | Niendorfer Gehege | 3.24 | 274 | 6 | 61 | 56 | 33.43 | 37.20 | 2.62 | 2.69 | 8 | 5 |
|  | Ohmoor | 1.55 | 187 | 3 | 62 | 56 | 50.05 | 41.70 | 3.34 | 3.09 | 10 | 7 |
|  | Stadtpark | 1.91 | 35 | 0 | 18 | 17 | 35.61 | 30.76 | 4.11 | 5.36 | 11 | 12 |
| NB (2016) | Bornmoor | 1.33 | 81 | ‑ | 38 | 43 | 25.53 | 28.05 | 2.42 | 3.11 | 13 | 5 |
|  | Borsteler Jäger | 3.25 | 70 | ‑ | 29 | 41 | 40.69 | 34.61 | 4.73 | 4.07 | 10 | 12 |
|  | Fischbeker Heide | 0.06 | (3) ^d^ | ‑ | ‑ | ‑ | ‑ | ‑ | ‑ | ‑ | ‑ | ‑ |
|  | Marienhöhe | 17.67 | 5 | ‑ | 2 | 3 | 25.50 | 1.00 | 9.50 | 0.00 | 0 | 100 |
|  | Meyers Park | 9.46 | 16 | ‑ | 7 | 9 | 28.29 | 26.44 | 9.08 | 6.44 | 29 | 11 |
|  | Niendorfer Gehege | 6.19 | 24 | ‑ | 14 | 10 | 34.86 | 42.20 | 6.31 | 11.20 | 7 | 10 |
|  | Ohmoor | 2.00 | 69 | ‑ | 34 | 35 | 34.71 | 43.09 | 4.39 | 5.01 | 12 | 6 |
|  | Stadtpark | 0.38 | 30 | ‑ | 8 | 22 | 31.88 | 43.91 | 5.33 | 5.26 | 0 | 5 |
| PO (2015) | Bornmoor | 0.14 | 2 | 0 | 1 | 1 | 19.00 | 12.00 | - | - | 0 | 0 |
|  | Borsteler Jäger | 1.50 | 243 | 5 | 50 | 85 | 26.98 | 21.62 | 2.42 | 1.78 | 18 | 22 |
|  | Fischbeker Heide | 3.11 | 329 | 6 | 127 | 133 | 17.83 | 16.08 | 1.15 | 0.92 | 23 | 23 |
|  | Marienhöhe | 1.28 | 595 | 30 | 77 | 57 | 19.16 | 15.12 | 1.88 | 1.25 | 34 | 28 |
|  | Meyers Park | 0.69 | 241 | 9 | 77 | 67 | 21.16 | 17.99 | 2.18 | 1.88 | 31 | 30 |
|  | Niendorfer Gehege | 3.59 | 627 | 18 | 131 | 109 | 22.90 | 14.67 | 1.65 | 1.12 | 25 | 22 |
|  | Ohmoor | 2.30 | 148 | 2 | 46 | 27 | 31.46 | 21.93 | 2.46 | 2.14 | 4 | 7 |
|  | Stadtpark | 0.73 | 73 | 3 | 44 | 28 | 30.64 | 20.89 | 2.92 | 3.60 | 11 | 18 |
| PO (2016) | Bornmoor | 2.07 | 1 | ‑ | 1 | 0 | 1.00 | ‑ | ‑ | ‑ | 0 | ‑ |
|  | Borsteler Jäger | 2.22 | 20 | ‑ | 11 | 9 | 20.09 | 7.33 | 5.58 | 2.60 | 18 | 44 |
|  | Fischbeker Heide | 1.65 | 70 | ‑ | 26 | 44 | 14.62 | 13.11 | 2.22 | 2.18 | 27 | 23 |
|  | Marienhöhe | 2.60 | 126 | ‑ | 64 | 62 | 12.31 | 8.84 | 1.46 | 1.14 | 17 | 23 |
|  | Meyers Park | 0.20 | 96 | ‑ | 51 | 45 | 13.98 | 10.64 | 2.92 | 2.79 | 31 | 42 |
|  | Niendorfer Gehege | 2.99 | 93 | ‑ | 44 | 49 | 15.43 | 10.57 | 2.46 | 1.48 | 20 | 33 |
|  | Ohmoor | 1.10 | 31 | ‑ | 16 | 15 | 18.81 | 14.20 | 4.97 | 3.88 | 6 | 20 |
|  | Stadtpark | 0.46 | 35 | ‑ | 12 | 23 | 34.67 | 12.78 | 9.89 | 2.53 | 25 | 26 |
| Total |  |  |  |  | 1589 | 1327 |  |  |  |  | 18.9 | 24.2 |

AP, *Abax parallelepipedus;* CN, *Carabus nemoralis;* NB, *Nebria brevicollis;* PO, *Pterostichus oblongopunctatus.* -, not applicable. ^a^ Density calculated from extra pitfall traps placed roughly 50 m away from trapping sites of this study and opened during the study period. Numbers indicate mean numbers of individuals per trap over 10 trapping days. ^b^ Please note not all individuals that were trapped were behavioural tested. ^c^ Number of individuals that were behavioural tested. ^d^ Misidentification possible due to occurrence of *Nebria salina* in this site. Individuals not taken for analyses.
