# Supplementary material for: Ground beetles in city forests: does urbanization predict a personality trait?
Source: PeerJ. 2018 Feb 20;6:e4360. doi: 10.7717/peerj.4360 (PMC5824674; doi:10.7717/peerj.4360)
Supplement: Table S3 — In the field each individual was tested once; in the laboratory each individual was tested twice. Coefficients (coeff.) in brackets: coefficients of non-significant terms just before dropping the terms, other coefficients (not in brackets): from minimal adequate model (note coefficients in brackets cannot be compared to coefficients from the minimal adequate models, since the simplification alters coefficients); bold p-value denotes significant effect; coefficients for factor levels give the difference to the reference level. [file peerj-06-4360-s004.docx]

| Species (data) | Random term | Variance | Fixed effect | Coeff. ^a^ | *Χ*^2^ | *DF* | *P* | Transf. | *N*_trials_ (*N*_ID_) |
| --- | --- | --- | --- | --- | --- | --- | --- | --- | --- |
| AP | Observer | 0 | (Mean) | 52.66 |  |  |  | none | 239 |
| (Field | Site | 123.89 | Thanatosis [yes] : Sex [female] | (-0.46) | < 0.01 | 1 | 0.9610 |  | (239) |
| 2015) | Week | 39.53 | Thanatosis [yes] | -16.09 | 13.19 | 1 | **0.0003** |  |  |
|  | (Residual) | 820.72 | Sex [female] | (-6.92) | 3.26 | 1 | 0.0709 |  |  |
| CN | Observer | 0.18 | (Mean) | 3.45 |  |  |  | sqrt | 321 |
| (Field | Site | 0.11 | Thanatosis [yes] : Sex [female] | (-0.84) | 3.31 | 1 | 0.0691 |  | (321) |
| 2015) | Week | 0.03 | Thanatosis [yes] | (-0.32) | 2.10 | 1 | 0.1474 |  |  |
|  | (Residual) | 2.93 | Sex [female] | -0.69 | 8.61 | 1 | **0.0033** |  |  |
| NB | Observer | 9.92 | (Mean) | 34.76 |  |  |  | none | 864 |
| (Field | Site | 18.31 | Thanatosis [yes] : Sex [female] | (4.18) | 0.82 | 1 | 0.3657 |  | (864) |
| 2015 + | Week | 62.45 | Thanatosis [yes] | -18.62 | 57.98 | 1 | **<0.0001** |  |  |
| 2016) | (Residual) | 446.53 | Sex [female] | (1.17) | 0.64 | 1 | 0.4250 |  |  |
| PO | Observer | 0.13 | (Mean) | 4.23 |  |  |  | sqrt | 1532 |
| (Field | Site | 0.15 | Thanatosis [yes] : Sex [female] | (0.14) | 0.48 | 1 | 0.4891 |  | (1532) |
| 2015 + | Week | 0.12 | Thanatosis [yes] | -1.29 | 135.20 | 1 | **<0.0001** |  |  |
| 2016) | (Residual) | 2.95 | Sex [female] | -0.53 | 34.28 | 1 | **<0.0001** |  |  |
| NB | ID | 199.36 | (Mean) | 57.86 |  |  |  | none | 590 |
| (Lab | Observer | 0 | Thanatosis [yes] : Sex [female] | (-11.99) | 0.65 | 1 | 0.4219 |  | (295) |
| 2016) | Site | 29.92 | Thanatosis [yes] | -32.14 | 19.23 | 1 | **<0.0001** |  |  |
|  | Week | 91.53 | Sex [female] | (-1.82) | 0.33 | 1 | 0.5641 |  |  |
|  | (Residual) | 915.60 |  |  |  |  |  |  |  |
| PO | ID | 1.09 | (Mean) | 6.06 |  |  |  | sqrt | 944 |
| (Lab | Observer | 0.06 | Thanatosis [yes] : Sex [female] | (-0.75) | 0.78 | 1 | 0.3767 |  | (472) |
| 2016) | Site | 0.02 | Thanatosis [yes] | -1.69 | 15.76 | 1 | **<0.0001** |  |  |
|  | Week | 0.31 | Sex [female] | (-0.30) | 3.39 | 1 | 0.0657 |  |  |
|  | (Residual) | 4.18 |  |  |  |  |  |  |  |

AP, *Abax parallelepipedus;* CN, *Carabus nemoralis;* NB, *Nebria brevicollis;* PO, *Pterostichus oblongopunctatus*; transf., transformation of response (sqrt, square-root taken). ^a^ Please note that coefficients are not back-transformed for those analyses in which response was transformed.
