# Supplementary material for: Ground beetles in city forests: does urbanization predict a personality trait?
Source: PeerJ. 2018 Feb 20;6:e4360. doi: 10.7717/peerj.4360 (PMC5824674; doi:10.7717/peerj.4360)
Supplement: Table S4 — For data including field tests adjusted repeatabilities were calculated with ambient temperature (temperature and temperature2) as fixed term. Bold test statistics denote significance. [file peerj-06-4360-s005.docx]

| Species | Data | *N*_trials_/N_ID_ | Method | *r* or *R*_s_ [95% CI] | Transf. |
| --- | --- | --- | --- | --- | --- |
| NB | field - l1 | 590/295 | adj repeatability | **0.152 [0.043; 0.268]** | sqrt |
|  |  |  | Spearman | **0.271 [0.157; 0.388]** | none |
|  | l1 - l2 | 590/295 | repeatability | **0.277 [0.172; 0.389]** | none |
|  |  |  | Spearman | **0.282 [0.167; 0.393]** | none |
|  | l1 - l2 female | 326/163 | repeatability | **0.249 [0.093; 0.386]** | none |
|  |  |  | Spearman | **0.260 [0.089; 0.413]** | none |
|  | l1 - l2 male | 264/132 | repeatability | **0.321 [0.152; 0.457]** | none |
|  |  |  | Spearman | **0.307 [0.141; 0.454]** | none |
|  | l1 - l2 UL high | 488/244 | repeatability | **0.320 [0.203; 0.422]** | none |
|  |  |  | Spearman | **0.326 [0.203; 0.437]** | none |
|  | l1 - l2 UL low | 102/51 | repeatability | 0.081 [0; 0.364] | none |
|  |  |  | Spearman | 0.072 [-0.217; 0.369] | none |
| PO | field - l1 | 944/472 | adj repeatability | 0.093 [0; 0.182] | sqrt |
|  |  |  | Spearman | **0.143 [0.047; 0.224]** | none |
|  | l1 - l2 | 944/472 | repeatability | **0.272 [0.181; 0.353]** | none |
|  |  |  | Spearman | **0.277 [0.181; 0.369]** | none |
|  | l1 - l2 female | 494/247 | repeatability | **0.271 [0.154; 0.382]** | none |
|  |  |  | Spearman | **0.263 [0.131; 0.384]** | none |
|  | l1 - l2 male | 450/225 | repeatability | **0.247 [0.123; 0.367]** | sqrt |
|  |  |  | Spearman | **0.284 [0.148; 0.417]** | none |
|  | l1 - l2 UL high | 290/145 | repeatability | **0.246 [0.098; 0.389]** | sqrt |
|  |  |  | Spearman | **0.243 [0.083; 0.399]** | none |
|  | l1 - l2 UL low | 654/327 | repeatability | **0.230 [0.135; 0.332]** | sqrt |
|  |  |  | Spearman | **0.289 [0.174; 0.400]** | none |

Field: field test, l1: test in laboratory at day 2, l2: test in laboratory at day 7; NB, *Nebria brevicollis;* PO, *Pterostichus oblongopunctatus*; CI, 95% confidence interval; CI was calculated using 1000 bootstraps; transf., transformation of response (sqrt, square-root taken); UL, urbanization level.
